# Functional recovery outcomes following acute stroke is associated with abundance of gut microbiota related to inflammation, butyrate and secondary bile acid

**DOI:** 10.3389/fresc.2022.1017180

**Published:** 2022-10-26

**Authors:** Tyler C. Hammond, Elizabeth Powell, Stefan J. Green, George Chlipala, Jacqueline Frank, Andrew T. Yackzan, Lucille M. Yanckello, Ya-Hsuan Chang, Xin Xing, Sally Heil, Joe E. Springer, Keith Pennypacker, Arnold Stromberg, Lumy Sawaki, Ai-Ling Lin

**Affiliations:** ^1^ Sanders-Brown Center on Aging, University of Kentucky, Lexington, KY, United States; ^2^ Department of Neuroscience, University of Kentucky, Lexington, KY, United States; ^3^ Department of Physical Medicine and Rehabilitation, University of Kentucky, Lexington, KY, United States; ^4^ Genomics and Microbiome Core Facility, Rush University, Chicago, IL, United States; ^5^ Research Informatics Core, University of Illinois Chicago, Chicago, IL, United States; ^6^ Center for Advanced Stroke Science, Department of Neurology, University of Kentucky, Lexington, KY, United States; ^7^ Department of Pharmacology and Nutritional Sciences, University of Kentucky, Lexington, KY, United States; ^8^ Department of Computer Science, University of Kentucky, Lexington, KY, United States; ^9^ School of Medicine, University of Missouri, Columbia, MO, United States; ^10^ Spinal Cord and Brain Injury Research Center, University of Kentucky, Lexington, KY, United States; ^11^ Department of Statistics, University of Kentucky, Lexington, KY, United States; ^12^ Department of Radiology, University of Missouri, Columbia, MO, United States; ^13^ Institute for Data Science & Informatics, University of Missouri, Columbia, MO United States; ^14^ Department of Biological Sciences, University of Missouri, Columbia, MO, United States

**Keywords:** stroke, microbiome, functional recovery, NIH toolbox, butyrate, secondary bile acid, leaky gut, dietary questionnaire

## Abstract

Accumulating evidence suggests that gut microbes modulate brain plasticity *via* the bidirectional gut-brain axis and play a role in stroke rehabilitation. However, the microbial species alterations associated with stroke and their correlation with functional outcome measures following acute stroke remain unknown. Here we measure post-stroke gut dysbiosis and how it correlates with gut permeability and cognitive functions in 12 stroke participants, 18 controls with risk factors for stroke, and 12 controls without risk factors. Stool samples were used to measure the microbiome with whole genome shotgun sequencing and leaky gut markers. We genotyped APOE status and measured diet composition and motor, cognitive, and emotional status using NIH Toolbox. We used linear regression methods to identify gut microbial associations with cognitive and emotional assessments. We did not find significance differences between the two control groups. In contrast, the bacteria populations of the Stroke group were statistically dissimilar from the control groups. Relative abundance analysis revealed notable decreases in butyrate-producing microbial taxa, secondary bile acid-producing taxa, and equol-producing taxa. The Stroke group had higher levels of the leaky gut marker alpha-1-antitrypsin in the stool than either of the groups and several taxa including *Roseburia* species (a butyrate producer) were negatively correlated with alpha-1-antitrypsin. Stroke participants scored lower on memory testing than those in the two control groups. Stroke participants with more *Roseburia* performed better on the picture vocabulary task; more *Bacteroides uniformis* (a butyrate producer) and less *Escherichia coli* (a pro-inflammatory species) reported higher levels of self-efficacy. Intakes of fiber, fruit and vegetable were lower, but sweetened beverages were higher, in the Stroke group compared with controls. Vegetable consumption was correlated with many bacterial changes among the participants, but only the species *Clostridium bolteae,* a pro-inflammatory species, was significantly associated with stroke. Our findings indicate that stroke is associated with a higher abundance of proinflammatory species and a lower abundance of butyrate producers and secondary bile acid producers. These altered microbial communities are associated with poorer functional performances. Future studies targeting the gut microbiome should be developed to elucidate whether its manipulation could optimize rehabilitation and boost recovery.

## Introduction

Over 795,000 people suffer a stroke every year in the United States alone (1). Recent advances in Stroke therapies have lowered stroke mortality, but survivors are often left severely impaired (2). Rehabilitation therapies such as physical therapy, occupational therapy, and speech therapy are beneficial for inducing neuroplasticity to overcome these impairments (3), but over 40% of stroke survivors are left with moderate to severe disabilities that markedly reduce quality of life (4). Novel multimodal approaches are needed to promote plasticity and restore sensorimotor function through a combination of current rehabilitation therapies with other treatments designed to foster neuroplasticity.

Accumulating evidence from animal studies suggests that gut microbes modulate brain plasticity *via* the bidirectional gut-brain axis and may play a role in functional recovery after stroke (5). A severely imbalanced microbial community, or dysbiosis, has been shown to occur following stroke, causing a systemic flood of neuro- and immunomodulatory substances due to increased gut permeability and decreased gut motility (6). These substances can impact neuroinflammation as commensal bacteria invade the bloodstream and as intestinal lymphocytes migrate from gut-associated lymphoid tissue to the brain (7). Fecal microbiota transplant has been shown to normalize brain lesion-induced dysbiosis and to improve stroke outcome in mice (7). The microbiome is modifiable as it is influenced by environmental factors such as diet and exercise and could potentially be an additional target in stroke rehabilitation through nutritional and pharmacological interventions (8, 9). Though it is unknown whether the findings from the bidirectional gut-brain axis in animals translate the same way into humans. Human studies thus far have suggested that gut dysbiosis occurs shortly following stroke at one time point and that this dysbiosis is associated with increased blood Apolipoprotein E (10) and IL-6 (11), decreased blood Trimethylamine-N-Oxide (12) and high-density lipoprotein (13), poor early functional outcomes (14), and 180-day mortality (15).

Currently, no human studies have been analyzed changes in the microbiome over the first three-week course of stroke rehabilitation and whether these changes correlate with gut permeability and subsequent recovery as measured by functional outcome measures. Furthermore, no human studies have included control groups with and without risk factors for stroke to delineate how the underlying risk factors contribute to microbiome differences. The goal of the study is to fill the gap by documenting the gut microbiome changes that occur following scute stroke in the first three weeks of rehabilitation and their associations with functional recovery measures. We also included two age-matched control groups with and without stroke risk factors, respectively, which allow us to inquire whether gut-brain axis changes can be attributed to the risk factors underlying stroke.

## Materials and methods

### Participants

All research activities were approved by the Institutional Review Board at the University of Kentucky. We recruited participants aged 55–85 for this study with consideration the population distribution of Kentucky. We recruited 12 patients in sub-stroke rehabilitation care after first time ischemic stroke Participants with clinically significant (unresolved, requiring on-going medical management or medication) pulmonary, gastrointestinal, dermatologic, hepatic, or renal functional abnormality were excluded. Individuals who had major gastrointestinal surgery within the past five years were also excluded.

Healthy participants (*N* = 30) were recruited through researchmatch.org and from advertisements posted by the Center for Clinical and Translational Science at the University of Kentucky. Among these participants, 18 did not have a history of stroke but have one or more common cardiovascular risk factors for stroke (Healthy group), and 12 did not have a history of stroke or common cardiovascular risk factors for stroke (At-Risk group) Participants followed the same inclusion/exclusion criteria listed above except for stroke. Table 1 describes the basic demographic characteristics of the participants. Those in the Stroke were slightly older than those in either control group. The Stroke and Healthy group had over 20% APOE ε2 carriers while the At-Risk group contained none. The participants in the Stroke were less educated than those in the Healthy and At-Risk groups. The Healthy group had a lower BMI than the Stroke group and the At-Risk group. The Healthy group by definition had no participants with diabetes, hypertension, or hyperlipidemia. In the stroke group, 66.7% of patients had a stroke in MCA territory, 8.3% in ACA territory, and 25.0% in subcortical territory.

**Table 1:** Participant characteristics.

	Stroke	At-risk	Healthy	*p*-value
*N*	12	18	12	
Age	68.5 ± 12.68	66.33 ± 6.53	64.75 ± 4.75	0.55
Sex (% Female)	83.33%	77.78%	91.67%	0.87
Race (% White)	100.00%	88.89%	91.67%	0.35
(% Black)	0.00%	11.11%	0.00%	0.35
(% Asian)	0.00%	0.00%	8.33%	0.35
Genotype (% APOE *ε*3/ε3)	41.67%	61.11%	41.47%	0.16
(% APOE ε3/ε4)	33.33%	33.33%	25.00%	0.16
(% APOE ε4/ε4)	0.00%	5.56%	0.00%	0.16
(% APOE ε2/ε4)	8.33%	0.00%	0.00%	0.16
(% APOE ε2/ε3)	16.67%	0.00%	33.33%	0.16
Education (years)	13.27 ± 2.97	16.78 ± 1.52	17.92 ± 2.07	<0.0001
BMI	29.65 ± 7.78	28.43 ± 5.81	24.72 ± 3.95	0.19
Diabetes	33.33%	16.67%	0%	0.08
Hypertension	75%	72.22%	0%	<0.0001
Hyperlipidemia	81.82%	50.00%	0%	<0.0001
Site of stroke (MCA)	66.7%			
(ACA)	8.3%			
(Subcortical)	25.0%			

Values are mean ± SD.

### Study design

Each individual received a verbal and written explanation of the purposes, procedures, and potential hazards of the study, and written consent was obtained. The University of California, San Diego Brief Assessment of Capacity to Consent (UBACC) was used to ensure decisional capacity then signed consent was obtained. Subjects were free to withdraw from the study at any time. This research had minimal risk. Following consent, study personnel collected the following information:

• Medical History: We obtained past medical history of the stroke patients from the electronic health record and questionnaires. These data were used solely for research purposes. Variables including age, gender, racial/ethnic background, education level, history of conventional vascular risk factors (hypertension, diabetes mellitus, atrial fibrillation, hyperlipoproteinemia, and smoking habit), and treatment during the acute phase were recorded and used as covariates for our analyses.
• Food Frequency: We assessed diet history using the Dietary Screener Questionnaire in the National Health and Nutrition Examination Survey for the dietary intake over the last month. (https://epi.grants.cancer.gov/nhanes/dietscreen/questionnaires.html). We included the results of the estimated intake of fiber, calcium, whole grains, sugar, dairy, fruits and vegetables, and sugar sweetened beverages.
• Oral Swab Sample: Oral swab was used to determine APOE genotype.
• Self-Care CARE Items: Section GG Self-Care Items are routinely used in the clinical care of inpatient stroke rehab patients to measure functional recovery.
• NIH Toolbox: Cognitive, Motor, Emotional, and Sensation measures from the NIH Toolbox were performed.
• Stool Samples: Stool samples were collected, genotyped, and analyzed using methods obtained from the International Human Microbiome Standards consortium (www.microbiome-standards.org) to determine gut microbial biodiversity.

The medical history, food frequency, NIH Toolbox measures, and stool samples were collected at admission and at a discharge visit for the stroke participants and at a three-month follow-up visit. For Healthy and At-Risk groups, these data were collected at admission and at a three-month follow-up visit. Due to the COVID-19 pandemic, many NIH Toolbox assessments were held *via* Zoom.

### Stool sample collection and analysis

Stool samples were collected in Zymo DNA stabilization solution with sarstedt feces tubes from feces catcher placed on toilet seat. Genomic DNA was extracted from 0.25 grams of stool using ZymoBIOMICS™ DNA Mini Kit and shipped to the Genomics and Microbiome core facility at Rush University for DNA quantification using fluorometer Qubit 3.0. Libraries were constructed and the PCR products purified using 1.0X speed beads and eluted in 15 ul of nuclease-free water and quantified by PicoGreen fluorometric assay (100X final dilution). The libraries were pooled and loaded onto a high sensitivity chip run on the Caliper LabChipGX (Perkin Elmer, Waltham, MA) for size estimation and sequenced using Illumina NextSeq/HiSeq platform. Unassembled sequencing reads were analyzed by the Research Informatics Core at the University of Illinois Chicago for microbiome analysis. Alpha diversity, beta diversity, and relative abundance counts were calculated. For alpha diversity, raw counts were rarefied to 2000 k and the Shannon diversity index was calculated using the vegan R package. For beta diversity, the Bray-Curtis dissimilarity index was used and a Principal Component Analysis (PCA) plot was generated to visualize the diversity. We used MetaPhlAn (Metagenomic Phylogenetic Analysis) to profile the composition of microbial communities using unique clade-specific marker genes identified from ∼17,000 reference genomes (∼13,500 bacterial and archaeal, ∼3,500 viral, and ∼110 eukaryotic) (16).

### APOE genotyping

We collected oral swabs from all participants and placed them in Zymo DNA stabilization solution (https://www.zymoresearch.com/collections/swab-collection/products/dna-rna-shield-collection-tube-w-swab). We sent the oral swabs to the Research Informatics Core at the University of Illinois Chicago for DNA extraction and amplification. The Core performed PCR to amplify and measure SNPs rs429358 and rs7412 that define the common allelic variants of Apolipoprotein E.

### Functional assessment

Functional analysis was measured using the Self-Care CARE Assessment from Section GG of the standardized patient assessment data elements in the following domains: Eating, Oral Hygiene, Toileting Hygiene, Shower/Bathe Self, Upper Body Dressing, Lower Body Dressing, Putting on/Taking off Footwear (17). Stroke patients were graded on these domains for how independently they were able to perform them on a scale from 1 to 6, with 1 being dependent and 6 being independent.

### NIH toolbox

Cognitive, Emotional, and Sensation function were measured using assessments from the NIH Toolbox. The picture vocabulary test was used to measure long-term or crystallized memory (18). The list sorting test was used to measure short-term memory, attention, and executive function (19). The sadness (20), meaning and purpose (21), self-efficacy (22), and support (23) questionnaires were used to measure self-reported values of these emotional domains. The pain intensity scale (24) was used to measure sensation.

### Statistical analyses

All statistical analyses were completed using JMP Statistical Software (SAS, Cary, NC, USA) and R Statistical Software (25). Two-sample t-test and 2-way ANOVA were used to determine differences between groups. The MaAsLin2 R package was used to normalize all variables and employ linear regression analysis to correlate various variables with microbiome measures (26). A false discovery rate of *q* < 0.25 was used in selecting significant variables to correct for multiple comparisons.

## Results

### Stroke patients had altered microbiome diversity and composition

**Figure 1:**
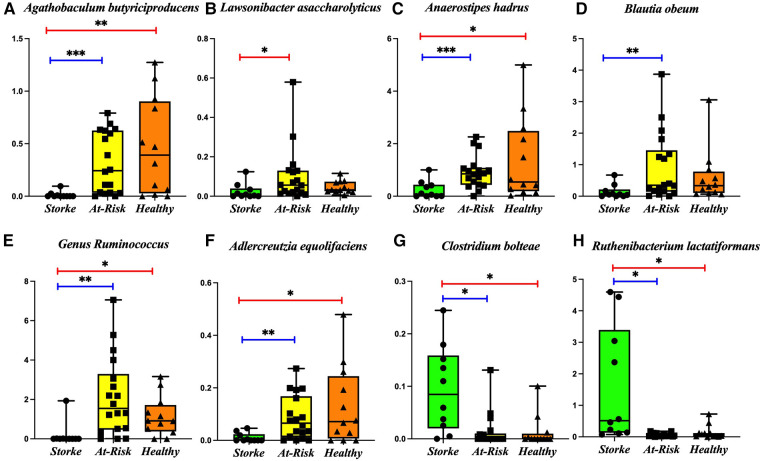
Bacterial taxa significantly changed in the stroke as compared to At-risk and healthy groups. The following taxa are lower in the stroke group: (**A**) *Agathobaculum butyriciproducens*, (**B**) *Lawsonibacter asaccharolyticus*, (**C**) *Anaerostipes hadrus*, (**D**) *Blautia obeum*, (**E**) Genus *Ruminococcus*, (**F**) *Adlercreutzia equolifaciens*, The following taxa are higher in the stroke group: (**G**) *Clostridium bolteae*, (**H**) *Ruthenibacterium lactatiformans*. *p-value < 0.05; **p-value < 0.01; ***p-value < 0.001.

We determined the beta diversity of the various groups using the Bray-Curtis dissimilarity index. An ANOSIM R test reveals a significant dissimilarity between the Stroke and At-Risk groups (R = 0.405, *p* = 0.001) and a significant dissimilarity between the Stroke and Healthy groups (R = 0.126, *p* = 0.039). The dissimilarity between the At-Risk and Healthy groups was not significant (R = 0.0381, *p* = 0.228). A detailed analysis of the microbial taxa shows significant decreases in butyrate producers [*Agathobaculum butyriciproducens, Lawsonibacter asaccharolyticus*, and *Anaerostipes hadrus* (Figures 1A–C)]*,* secondary bile acid producers *[Blautia obeum* and the genus *Ruminococcus* (Figures 1D,E)] and equol producers [*Adlercreutzia equolifaciens* (Figure 1F)]*.* There was a significant increase in a couple of pro-inflammatory taxa, including *Clostridium bolteae* and *Ruthenibacterium lactatiformans*, (Figures 1G,H).

### Stroke dysbiosis is associated with leaky gut markers

**Figure 2:**
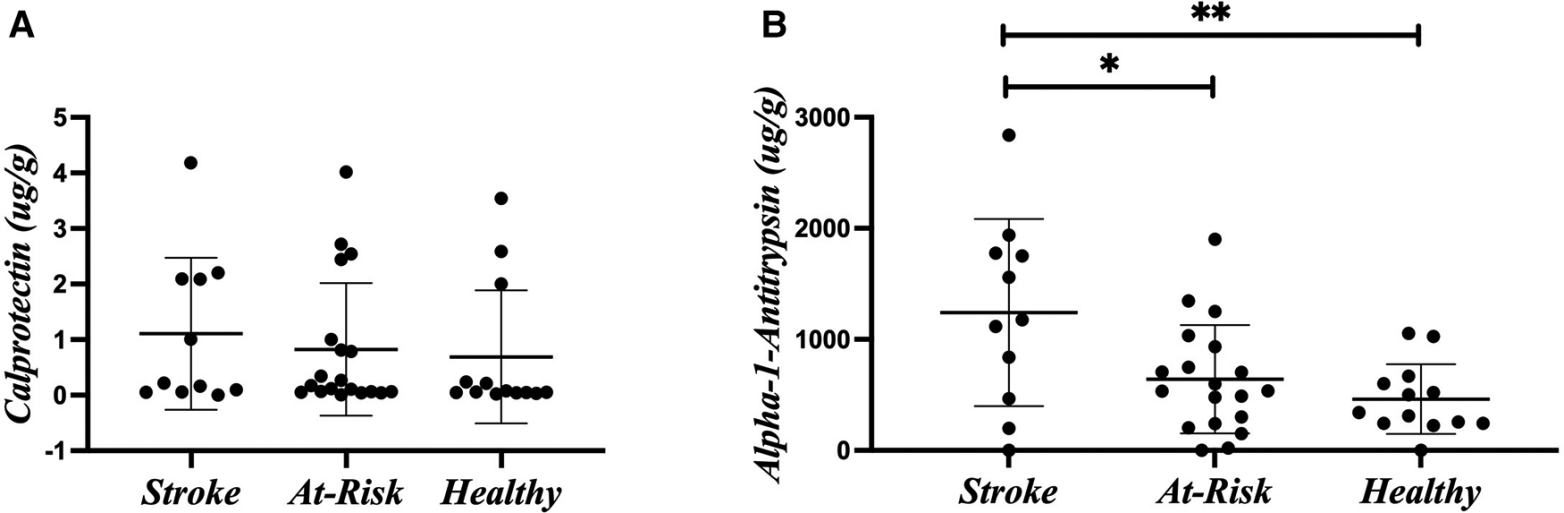
Leaky gut markers. (**A**) Calprotectin. (**B**) Alpha-1-antitrypsin. Leaky gut markers were compared amongst the participant groups using Kruskal-Wallis Test. *p-value < 0.05; **p-value < 0.01.

**Figure 3:**
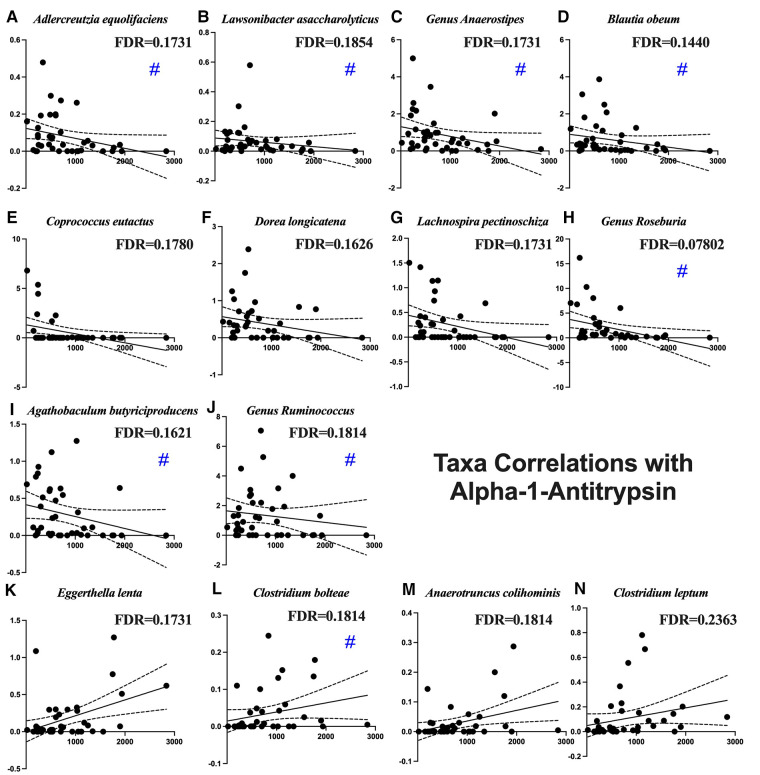
Microbial taxa associated with fecal alpha-1-antitrypsin. Blue pound signs indicate bacteria which were also associated with stroke. Negative associations include (**A**) *Adlercreutzia equolifaciens*, (**B**) *Lawsonibacter asaccharolyticus*, (**C**) the genus *Anaerostipes*, (**D**) *Blautia obeum*, (**E**) *Coprococcus eutactus*, (**F**) *Dorea longicatena*, (**G**) *Lachnospira pectinoschiza*, (**H**) the genus *Roseburia*, (**I**) *Agathobaculum butyriciproducens*, and (**J**) the genus *Ruminococcus*. Positive associations include (**K**) *Eggerthella lenta*, (**L**) *Clostridium bolteae*, (**M**) *Anaerotruncus colihominis*, and (**N**) *Clostridium leptum*.

Figure 2 shows the average of the leaky gut markers amongst the groups. The calprotectin assay is a marker for intestinal inflammation and did not show differences among the groups (Figure 2A). Alpha-1-antitrypsin is a marker for intestinal permeability and was significantly increased in the Stroke (Figure 2B). Table 2 shows the associations of alpha-1-antitrypsin with various microbial taxa. The presence of alpha-1-antitrypsin was inversely associated with several microbial taxa, including *Adlercreutzia equolifaciens*, *Lawsonibacter asaccharolyticus*, the genus *Anaerostipes*, *Blautia obeum*, *Coprococcus eutactus*, *Dorea longicatena*, *Lachnospira pectinoschiza*, the genus *Roseburia*, *Agathobaculum butyriciproducens*, and the genus *Ruminococcus* (Figures 3A–J). Alpha-1-antitrypsin was positively associated with *Eggerthella lenta*, *Clostridium bolteae*, *Anaerotruncus colihominis*, and *Clostridium leptum* (Figures 3K–N).

**Table 2:** Microbial taxa associated with fecal alpha-1-antitrypsin.

Bacteria	Coefficient	*Q*-value
**Taxa positively correlated with alpha-1-antitrypsin**		
* Eggerthella lenta*	0.468	0.1731
* Clostridium bolteae*	0.274	0.1814
* Clostridium leptum*	0.344	0.2363
* Anaerotruncus colihominis*	0.456	0.1814
**Taxa negatively correlated with alpha-1-antitrypsin**		
* Adlercreutzia equolifaciens*	−0.307	0.1731
* *Genus *Anaerostipes*	−0.298	0.1731
* *Family *Lachnospiraceae*	−0.113	0.2074
* Lachnospira pectinoschiza*	−0.215	0.1731
* *Genus *Roseburia*	−0.42	0.07802
* Roseburia inulinivorans*	−0.299	0.1731
* *Genus *Ruminococcus*	−0.457	0.1814
* Blautia obeum*	−0.359	0.1440
* Dorea longicatena*	−0.473	0.1626
* Lawsonibacter asaccharolyticus*	−0.256	0.1854

*Q*-value is calculated from false discovery rate.

### Bacteria are associated with dietary intake

**Figure 4:**
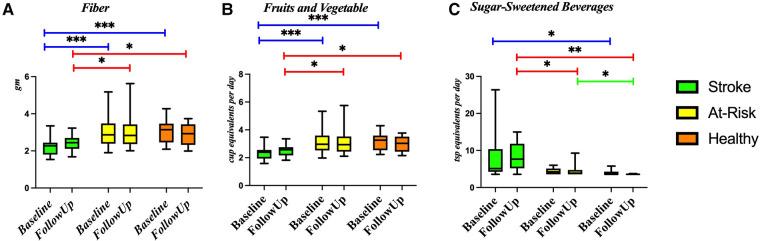
Diet composition amongst the groups. Diet was compared between each of the groups using a Wilcoxon rank sum test in several components: (**A**) fiber, (**B**) fruit and vegetables, and (**C**) sugar-sweetened beverages. *p-value < 0.05; **p-value < 0.01; ***p-value < 0.001.

**Figure 5:**
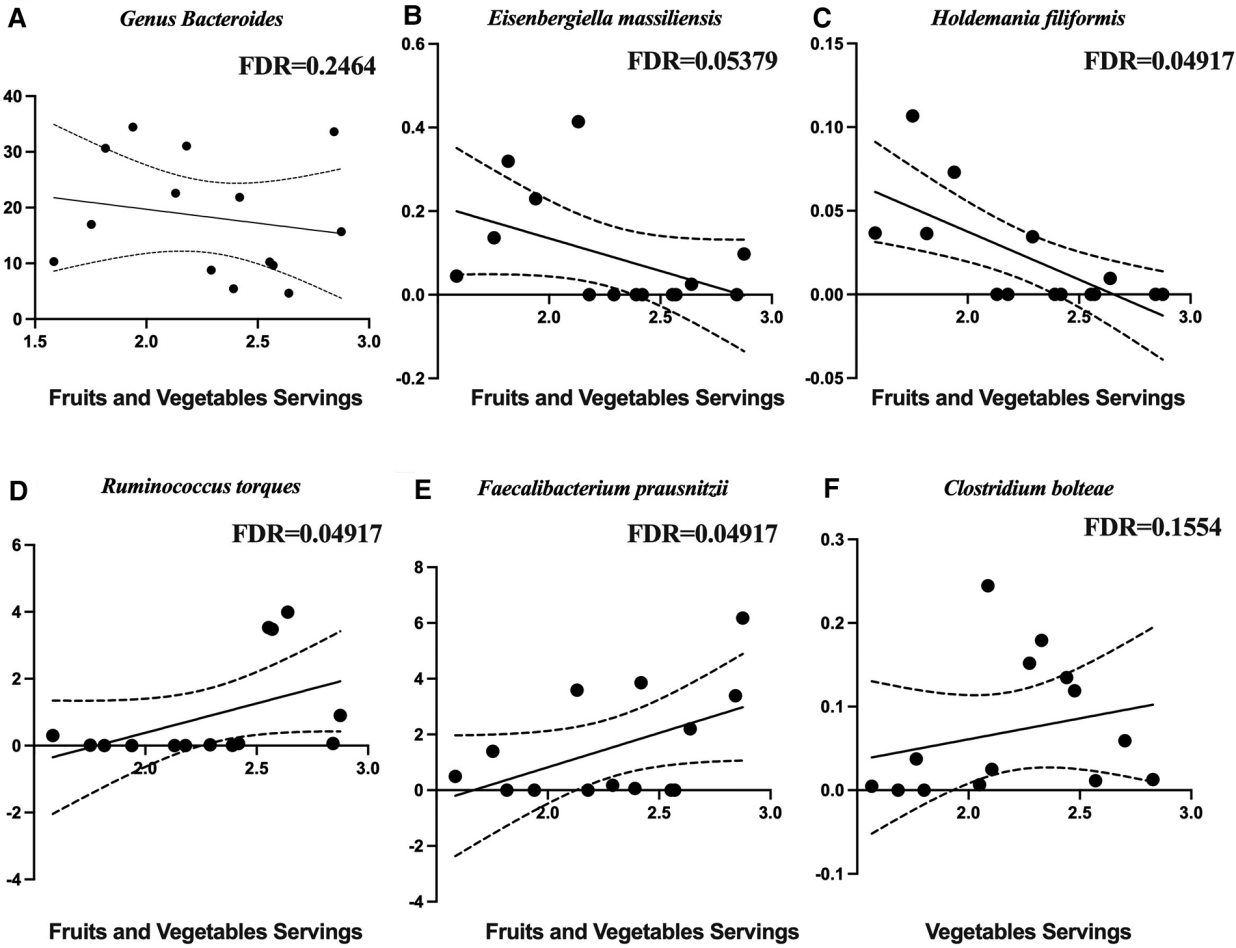
Microbial taxa associated with diet in the stroke participants. Negative associations with fruits and vegetables are with (**A**) the genus *Bacteroides*, (**B**) *Eisenbergiella massiliensis*, and (**C**) *Holdemania filiformis*. Positive associations with fruits and vegetables are with (**D**) *Ruminococcus torques*, (**E**) *Faecalibacterium prausnitzii*, and (**F**) *clostridium bolteae*.

Figure 4 shows the diet composition amongst the different participant groups. The Stroke group ate significantly less fiber (Figure 4A) and fruits and vegetables (Figure 4B) and more sugar sweetened beverages (Figure 4C). We correlated diet composition with microbial taxa in the Stroke group using linear correlation (Table 3). We found that an increase of fruits and vegetables was associated with a lower abundance of the genus *Bacteroides, Eisenbergiella massiliensis,* and *Holdemania filiformis* (Figures 5A–C) and a higher abundance of *Ruminococcus torques and Faecalibacterium prausnitzii* (Figures 5D,E). A higher intake of vegetables only was associated with an increase in the relative abundance of *Clostridium bolteae* (Figure 5F).

**Table 3:** Microbial taxa associated with diet in stroke participants.

Stroke diet feature	Microbial taxa	Coef	*Q*-value
Fiber	None		
Calcium	None		
Whole grains	None		
Sugar	None		
Dairy	None		
Fruits and vegetables	*Ruminococcus torques*	1.00	0.04917
	*Eisenbergiella massiliensis*	−0.301	0.05379
	*Faecalibacterium prausnitzii*	0.0974	0.04917
	*Holdemania filiformis*	−0.385	0.04917
	Genus *Bacteroides*	−0.153	0.2464
Vegetables only	*Clostridium bolteae*	0.578	0.1554
Sugar sweetened beverages	None		

#### Functional changes induced by stroke

**Figure 6:**
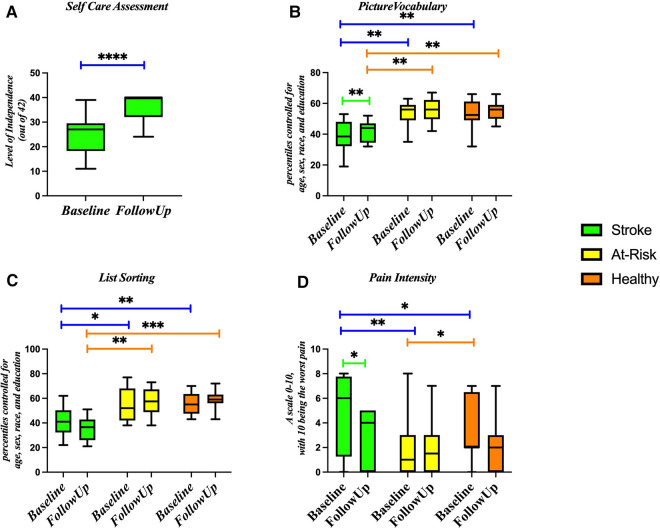
Markers of stroke function at baseline and follow up for stroke, at-risk, and healthy participants. Assessments include (**A**) self-care assessment, (**B**) picture vocabulary, (**C**) list sorting, and (**D**) pain intensity. *p-value < 0.05; **p-value < 0.01; ***p-value < 0.001.

On the Self Care Assessment, the Stroke group reported an average score of 25.4 out of 42 on admission to the hospital and an average score of 36.6 on discharge from the hospital (Figure 6A). On average, the Stroke group scored in the 35th percentile on the picture vocabulary test, the At-Risk group scored in the 52nd percentile, and the Healthy group scored in the 53rd percentile (Figure 6B). On the list sorting test, the Stroke group scored in the 38th percentile, the At-Risk group scored in the 52nd percentile, and the Healthy group scored in the 57th percentile (Figure 6C). On the pain questionnaire, the Stroke group reported a 4.9 out of 10, the At-Risk group reported a 2 out of 10, and the Healthy group reported a 3.25 out of 10 (Figure 6D).

### Microbiota are associated with markers of stroke recovery

Table 4 shows the correlations. *Collinsella aerofaciens* was positively correlated with self care scores**.** The genus *Roseburia* was positively correlated with scores on the picture vocabulary test for the Stroke group. *Bacteroides uniformis* and *Alistipes putredinis* were positively correlated with self-efficacy score and *Escherichia coli* was negatively correlated with self-efficacy. From the *Actinobacteria* phylum, the class *Coriobacteriia* is positively correlated with support. From the *Bacteroidetes* phylum, the family *Odoribacteraceae* is positively correlated with support. From the *Firmicutes* phylum, the genus *Eubacterium*, the family *Acidaminococcaceae*, *Roseburia intestinalis, and Phascolarctobacterium faecium* are positively correlated with support. From the *Bacteroidetes* phylum, *Bacteroides ovatus* is negatively correlated with support. From the *Firmicutes* phylum, *Erysipelatoclostridium ramosum* and *Flavonifractor plautii* were negatively correlated with support. From the *Proteobacteria* phylum, the family *Veillonellaceae* was negatively correlated with support. The family *Eubacteriaceae* was positively correlated with scores on the meaning and purpose questionnaire. In the Stroke group, *Alistipes shahii* was positively correlated with pain scores.

**Table 4:** Microbial taxa correlated with markers of stroke recovery.

Marker of stroke recovery	Bacteria	Coefficient	*Q*-value
Self-care assessment	*Collinsella aerofaciens*	0.772	0.01921
Picture vocabulary test	Genus *Roseburia*	0.593	0.1546
List sorting test	None		
Self-efficacy questionnaire	*Bacteroides uniformis*	0.337	0.2202
	Family *Enterobacteriaceae*	−0.766	0.1747
	*Escherichia coli*	−0.483	0.2202
Sadness questionnaire	None		
Meaning and purpose Questionnaire	Family *Eubacteriaceae*	1.29	0.1205
Support questionnaire	Class *Coriobacteriia*	0.299	0.1691
	Family *Odoribacteraceae*	0.215	0.1888
	Genus *Eubacterium*	0.870	0.2258
	Family *Acidaminococcaceae*	0.133	0.2258
	*Roseburia intestinalis*	0.0584	0.2258
	*Phascolarctobacterium faecium*	0.0505	0.1691
	*Bacteroides ovatus*	−0.238	0.1888
	*Erysipelatoclostridium ramosum*	−0.375	0.2258
	*Flavonifractor plautii*	−0.437	0.1580
	Family *Veillonellaceae*	−0.683	0.2258
Pain self-rating	*Alistipes shahii*	0.311	0.04039

## Discussion

Here we measured the gut microbiome in the first three weeks of rehabilitation following stroke and its associations with leaky gut markers, dietary intake, and functional recovery measures in 12 stroke participants, 18 control participants with risk factors for stroke, and 12 Healthy participants. We found significantly lower abundances of butyrate producers, secondary bile acid producers, equol producers, and sulfate reducers in the Stroke group and significantly higher abundances of pro-inflammatory taxa. We found no differences between with the At-Risk and Healhty groups, suggesting that the microbiome differences are associated with the stroke itself and not the underlying risk factors.

We found significant dissimilarity between the groups on beta diversity which is consistent with previous human studies that found high dissimilarity between ischemic stroke patients and healthy (27, 28). A detailed analysis of the relative abundance of the microbial taxa revealed several taxa that were lower in the Stroke group compared to either of the control groups. *Agathobaculum butyriciproducens* is a strictly anaerobic and butyric acid-producing bacteria that has had impressive success in restoring cognition in Alzheimer's disease mouse models (29). *Anaerostipes hadrus* can produce butyrate from carbohydrates or lactate (30, 31) and is often decreased in diabetes (32). *Eubacterium rectale* is also a butyrate producer (33) responsible for metabolizing dietary plant polysaccharides (34); it is increased in obesity (35) and reduces inflammatory dendritic cells (33). *Lawsonibacter asaccharolyticus* is also a butyrate producer (36). *Blautia obeum* is a natural producer of bile salt hydrolases (37) as well as lantibiotics that inhibit the growth of pathogenic bacteria (38); it has previously been shown to be decreased in acute cerebral infarction (39). *Roseburia* is a butyrate producer (40) that utilizes acetate (31) and increases serotonin and melatonin and is reduced in ulcerative colitis (41) and hypertension (42); treatment with *Roseburia hominis* in ulcerative colitis has been shown to strengthen gut barrier function and enhance T regulatory cells (43). *Ruminococcus* bacteria produce secondary bile acids (44). *Adlercreutzia equolifaciens* is an equol producer (45) and low abundances have been associated with primary sclerosing cholangitis (46). *Desulfovibrionaceae* reduces sulfate, a compound causing diarrhea and intestinal pain in overabundance (47).

Several bacterial taxa were significantly higher in the Stroke group as compared to the control groups. *Ruthenibacterium lactatiformans* is an obligate anaerobe that is a major lactate producer and is also found to be increased in patients with multiple sclerosis (48). *Clostridium bolteae* is an obligate anaerobe commonly found to be increased in patients with autism (49), neuromyelitis optica spectrum disorders (50), multiple sclerosis (51, 52), and spondyloarthritis (53). *Acidaminococcus intestini* has been associated with a pro-inflammatory diet (54). Increased abundance of the genus *Lachnoclostridium* is associated with ulcerative colitis (55) and obesity (56). The genus *Anaeromassilibacillus* is associated with malnutrition (57). These bacteria are all associated with negative outcomes prevalent in stroke.

Importantly, the Stroke contained a significant percentage of APOE ε2 carriers, which potentially shapes the gut microbiome characteristics for this group with a higher relative abundance of *Ruminococcaceae* and *Gemmiger* species and a lower abundance of *Prevotellaceae* species (58).

Alpha-1-antitrypsin can be a marker of increased intestinal permeability when it is found in the stool. Calprotectin can be a marker of intestinal inflammation. There was a large increase of fecal alpha-1-antitrypsin in our participants with stroke. Gut inflammatory and immune responses following stroke are central to this increased gut permeability. The bacteria that we found are disrupted following stroke that also correlate with alpha-1-antitrypsin include increased *Clostridium bolteae* and decreased *Adlercreutzia equolifaciens, Anaerostipes*, *Roseburia*, *Ruminococcus*, *Blautia obeum*, *Agathobaculum butyriciproducens*, and *Lawsonibacter asaccharolyticus*. *Roseburia* has consistently been associated with intestinal permeability both as a microbe that bolsters intestinal permeability (59) and as a microbe that changes in response to changes in the intestinal permeability (60–62). We did not see a significant increase in calprotectin, likely because it is less stable at room temperature (63).

We found that certain dietary features are associated with the abundance of specific bacteria. This is congruent with other groups who have found that dietary modification is associated with gut microbiome composition (64). In our study particularly, we found that most of the bacteria that were affected by stroke were not affected by diet, with the exception of a positive relationship between *Clostridium bolteae* and vegetable intake. This is interesting since *Clostridium bolteae* was increased in stroke. There are many studies that describe the effects of a vegetarian diet on the microbiome, noting its association with an increased prevalence of anti-inflammatory species (65, 66). It is possible that dietary elements could promote a microbiome favoring functional recovery following stroke.

Our results indicate that the participants performed functionally better at discharge than at admission to the hospital. This is expected in the rehab setting. The species *Collinsella aerofaciens* was positively correlated with the Self-Care Assessment. *Collinsella aerofaciens* is a natural bile salt hydrolase producer (37, 67) that is naturally increased in response to a cow milk supplemented diet (68, 69). While *Collinsella aerofaciens* is generally considered to be a pro-inflammatory species that increases gut permeability (70), it has been associated with healthy clinical outcomes (71). While bile acids do not normally cross the blood brain barrier, in the context of a leaky blood brain barrier, they can accumulate in the hypothalamus and inhibit the hypothalamic-pituitary-adrenal axis, thereby suppressing the inflammatory response (72, 73).

Participants in the Stroke group perform more poorly on cognitive tests compared to those in the control groups. Cognitive impairment is common following stroke and is often the precursor to dementia and cognitive decline (74). Amongst stroke participants, the genus *Roseburia* was positively correlated with performance on the picture vocabulary test. Previous groups have seen a correlation of memory performance with *Roseburia* (75, 76). It is possible that *Roseburia* enhances memory performance through butyrate production since butyrate has been shown to be a cognitive enhancer of a weak memory (77).

We found that the Stroke group reported lower self-efficacy than the control groups. *Bacteroides uniformis* and *Alistipes putredinis* were positively correlated with self-efficacy and *Escherichia coli* was negatively correlated with self-efficacy. The concept of self-efficacy encapsulates a person's perception of their capability for performance (78). Self-efficacy has been shown to be a strong variable in impacting recovery following stroke (79). While it is not known why these taxa correlate with self-efficacy, it is possible that individuals with a higher self-efficacy are more likely to make healthy choices following stroke which would correlate with butyrate producing bacteria like *Bacteroides uniformis* as opposed to inflammatory species like *Escherichia coli* (80). The *Enterobacteriaceae* family, which *E. coli* is from, has previously been reported as being associated with bad outcomes in the context of stroke (15, 81, 82).

The class *Coriobacteriia* was positively correlated with support. Social isolation stress has been shown to alter the gut-brain axis (83). The class *Coriobacteriia* contains many species which are equol producers. Since equol producers and butyrate producers are associated with social support, it is possible that one mechanism by which people who have strokes and who have more support do better is mediated by the microbiome.

The Stroke group reported experiencing more pain than the control groups. Pain is a very common phenomenon following stroke and can include complex regional pain syndrome, musculoskeletal pain, spasticity-related pain, and post-stroke headache (84). In the Stroke group, *Alistipes shahii* was positively associated with reported pain. The abundance of *Alistipes shahii* is highly positively correlated with trimethylamine-N-oxide (TMAO), a marker of poor cardiometabolic health (85). While it is unknown how *Alistipes shahii* is associated with pain, many other studies have linked the microbiome with pain (86), including other species of *Alistipes* (87).

While this study provides valuable insights into the associations of the composition of bacterial communities in the gut and various markers of stroke recovery, it has many limitations. As a prospective case control study, it cannot definitively say that the associations are causing stroke recovery to be altered. Future experiments should test the associations found here to determine causation. Further, we did not have access to cognitive status prior to stroke or baseline stroke severity due to the recruitment procedure feasibility. We acknowledge that these are major limitation as the previous cognitive status could influence the microbiome composition and performance on post-stroke cognitive assessments and the baseline stroke severity could impact potential for recovery. Additionally, our sample consists largely of older white adults from Kentucky. Larger studies comprising more diverse populations are needed to see whether these associations are generalizable. Furthermore, we used a false discovery rate of *q* < 0.25. This means that up to 25% of our found associations may be false positives. More targeted experiments are needed in the future to better characterize these associations.

Altogether, we found that stroke was associated with an increase of pro-inflammatory bacterial taxa and a decrease in taxa that produce butyrate and secondary bile acids necessary for healthy metabolic function. This shift towards inflammation is likely due to the activation of the sympathetic nervous system and hypothalamic-pituitary-adrenal axis in response to the stroke that increases gut permeability and decreases gut motility. While this inflammatory shift can help to mitigate the acute effects of stroke in the brain, the residual inflammation in the weeks and months following the stroke is likely undermining recovery. Previous studies have also found that butyrate-producing bacteria were significantly reduced in cerebral ischemia patients and that this reduction is associated with poor outcomes (27, 88, 89). Future studies should explore treatments targeting the composition of microbial communities following stroke as a way to boost recovery from stroke in combination with other rehabilitation therapies. It is possible that optimizing butyrate producers, secondary bile acid producers, equol producers, and sulfate reducers in the gut through dietary interventions (90–93) could contribute to creating a rehabilitation environment where recovery is boosted.

## Data Availability

The datasets presented in this study can be found in online repositories. The names of the repository/repositories and accession number(s) can be found below: https://www.ncbi.nlm.nih.gov/, Under review.
